# Evaluating the impact of genomic epidemiology of methicillin-resistant Staphylococcus aureus (MRSA) on hospital infection prevention and control decisions

**DOI:** 10.1099/mgen.0.001235

**Published:** 2024-04-17

**Authors:** Beth Blane, Kathy E. Raven, Nicholas M. Brown, Ewan M. Harrison, Francesc Coll, Rachel Thaxter, David A. Enoch, Theodore Gouliouris, Danielle Leek, Sophia T. Girgis, Asha Akram, Marta Matuszewska, Paul Rhodes, Julian Parkhill, Sharon J. Peacock

**Affiliations:** 1 Department of Medicine, University of Cambridge, Box 157 Addenbrooke’s Hospital, Hills Road, Cambridge, UK; 2 Clinical Microbiology and Public Health Laboratory, UK Health Security Agency, Addenbrooke’s Hospital, Cambridge, UK; 3 Wellcome Sanger Institute, Hinxton, Cambridge, UK; 4 Department of Public Health and Primary Care, University of Cambridge, Cambridge, UK; 5 Department of Infection Biology, Faculty of Infectious and Tropical Diseases, London School of Hygiene & Tropical Medicine, Keppel Street, London WC1E 7HT, UK; 6 Next Gen Diagnostics, LLC, (NGD) Mountain View, CA, USA; 7 Broers Building, 21 JJ Thomson Ave., Cambridge, UK; 8 Department of Veterinary Medicine, University of Cambridge, Madingley Road, Cambridge, UK

**Keywords:** genomic epidemiology, infection prevention and control, MRSA, outbreak, pseudo-outbreak, sequencing

## Abstract

Genomic epidemiology enhances the ability to detect and refute methicillin-resistant *Staphylococcus aureus* (MRSA) outbreaks in healthcare settings, but its routine introduction requires further evidence of benefits for patients and resource utilization. We performed a 12 month prospective study at Cambridge University Hospitals NHS Foundation Trust in the UK to capture its impact on hospital infection prevention and control (IPC) decisions. MRSA-positive samples were identified via the hospital microbiology laboratory between November 2018 and November 2019. We included samples from in-patients, clinic out-patients, people reviewed in the Emergency Department and healthcare workers screened by Occupational Health. We sequenced the first MRSA isolate from 823 consecutive individuals, defined their pairwise genetic relatedness, and sought epidemiological links in the hospital and community. Genomic analysis of 823 MRSA isolates identified 72 genetic clusters of two or more isolates containing 339/823 (41 %) of the cases. Epidemiological links were identified between two or more cases for 190 (23 %) individuals in 34/72 clusters. Weekly genomic epidemiology updates were shared with the IPC team, culminating in 49 face-to-face meetings and 21 written communications. Seventeen clusters were identified that were consistent with hospital MRSA transmission, discussion of which led to additional IPC actions in 14 of these. Two outbreaks were also identified where transmission had occurred in the community prior to hospital presentation; these were escalated to relevant IPC teams. We identified 38 instances where two or more in-patients shared a ward location on overlapping dates but carried unrelated MRSA isolates (pseudo-outbreaks); research data led to de-escalation of investigations in six of these. Our findings provide further support for the routine use of genomic epidemiology to enhance and target IPC resources.

## Data Summary

All genomic data generated and analysed during the current study are available from the European Nucleotide Archive (ENA) under study accession ERP155550 (see Table S1, available in the online version of this article, for additional details).

**Impact Statement**

Genomic epidemiology of healthcare-associated pathogens increases the accuracy of outbreak detection and can refute outbreaks when patients are epidemiologically linked but do not carry related isolates of the same species. However, uptake into routine infection prevention and control (IPC) practice has been slow, in part because of a paucity of data that quantify benefits to patients and resource utilization. We undertook a 12 month study in a UK teaching hospital to codify how provision of methicillin-resistant *Staphylococcus aureus* (MRSA) genomic epidemiological data to IPC teams influenced their decisions. We optimized cost by performing sequencing runs when 21 clinical MRSA isolates had accrued and used a fully automated analysis tool to compare MRSA genome relatedness. A rolling programme of genomic epidemiology updates were provided each week to IPC teams. Their decisions were classified based on a simple flow chart that described pathways to escalation and de-escalation of IPC activities. Detailed discussion explored 17 hospital outbreaks; genomic epidemiology data altered IPC actions in 14 of these. We identified 38 pseudo-outbreaks; IPC activities were de-scalated in six of these. The study focused on MRSA transmission in hospital and did not include MRSA-positive samples taken in the community, but we identified two significant outbreaks involving patients prior to hospital presentation. In conclusion, these findings add to the growing weight of evidence that genomic epidemiology of nosocomial pathogens is a valuable and impactful tool in routine healthcare practice.

## Introduction

Methicillin-resistant *Staphylococcus aureus* (MRSA) is a leading cause of death due to antimicrobial resistance worldwide [1]. The development of MRSA carriage and infection are frequently healthcare-associated, and preventing and detecting MRSA transmission is a key activity for infection prevention and control (IPC) teams. The adoption of genomic epidemiology where patient movement is combined with bacterial relatedness based on genome sequencing enhances the ability to detect and refute MRSA outbreaks [2,3]. This can be used to strengthen traditional hospital epidemiology, detect transmission links that may not be apparent with standard measures, and inform enhanced surveillance and a targeted IPC response. There is also evidence for the value of prospective genomic surveillance whereby all MRSA identified in the clinical laboratory are routinely sequenced and their genomes compared at the time of isolation [4,5], rather than once a suspected outbreak has occurred. However, support for the routine implementation of genomic epidemiology in hospitals requires evidence of measurable benefits for patient care and/or reductions in use of system resources.

Deriving the greatest benefit from genomic epidemiology is dependent upon access to fast and affordable sequencing. It is also necessary to have the capability to analyse sequence information in a system that can identify putative transmission for epidemiological validation and IPC-targeted interventions. The inherent high throughput and low cost requirements suggest the need for validated fully automated bioinformatic systems. Previously, we reported the early evaluation of an automated analysis tool for MRSA genomes [Next Gen Diagnostics (NGD) bioinformatics system] to determine pairwise relatedness and identify clusters, using a definition of ≤25 single nucleotide polymorphisms (SNPs) apart [6]. A second study demonstrated that the tool provided highly accurate predictions of antibiotic susceptibility to 31 agents [7]. Evaluation was extended to 781 MRSA genomes from 777 consecutive patients, which was compared with analytic outputs from a bespoke researcher-led informatics pipeline [8]. There was full concordance between the two analysis methods for species identification, detection of *mec* genes and multi-locus sequence type (ST) assignment. In total, 3311 isolate pairs ≤25 SNPs apart were identified by at least one method. These had a median (range) SNP difference between the two methods of 1.2 SNPs (0–22 SNPs), with most isolate pairs (87 %) varying by ≤2 SNPs. Both pipelines clustered 338 isolates/334 patients into 66 unique clusters based on genetic relatedness, with the automated system identifying four putative clusters not detected by the researcher-led pipeline [8].

These research studies show the feasibility of implementing MRSA sequencing to reduce transmission, and based on a mathematical modelling study of cost effectiveness, pro-active genomic surveillance of MRSA is likely to provide a positive cost–benefit ratio to the healthcare system [9]. Here, we describe a 12 month prospective study of MRSA sequencing in which genomic epidemiology supported by the NGD bioinformatics system was combined with weekly meetings with the IPC team to discuss the results and record decisions on interventional actions. This allowed the characterization of the benefit derived to IPC decisions and resource utilization from this means of transmission detection.

## Methods

### Study setting, patients and samples

A prospective observational cohort study was conducted at the Public Health England (PHE) (now referred to as the UK Health Security Agency) Clinical Microbiology and Public Health Laboratory at the Cambridge University Hospitals NHS Foundation Trust (CUH) in the UK. All hospital inpatients were routinely screened for MRSA on admission to hospital, and screening was repeated weekly in critical care units. Additional clinical specimens were taken as part of routine clinical care. Patients with a prolonged in-patient stay were rescreened on day 40. Compliance with the screening policy was audited on a monthly basis. Mean compliance over the 12 month period for elective admissions was 95 % (range 94–96 %) and for emergency admissions was 92 % (range 89–93 %). All individuals with MRSA-positive samples from any body site that were taken at any location at CUH during a 12 month period between November 2018 and November 2019 were included in the study. This included out-patients, in-patients, patients who were reviewed in the Emergency Department but not subsequently admitted, and hospital healthcare workers with samples submitted by the Occupational Health department. The study excluded samples submitted by General Practitioners (GPs) and other care providers in the community. MRSA-positive cases were identified using the hospital IT system [EPIC EMR (Hyperspace 2014; Epic Systems Corporation)].

Within the diagnostic laboratory, suspected MRSA was identified as *S. aureus* using MALDI-TOF MS (Brucker Corporation). Antimicrobial susceptibility testing was performed using the disc diffusion method (EUCAST). Putative or confirmed MRSA-positive culture plates were prospectively retrieved from the diagnostic laboratory by the research team and individual colonies confirmed as *S. aureus* using the Staph Latex kit (Pro-lab Diagnostics). Pure cultures were obtained by sub-culture of single colonies on Columbia blood agar and stored at -70 °C in Microbank vials. For each MRSA-positive patient, data were collected on specimen type (MRSA screen or a clinical sample), date and place of sampling, patient age, date of birth, any history of MRSA carriage, GP practice, postcode of normal residence, admission from a long-term care facility and outcome (discharge or in-hospital death). These data fields were not collected for healthcare workers. A unique study code was used to anonymize participant data, laboratory sample identifiers, MRSA isolates and genomes for each case.

### Sequencing

A sequencing run was performed when 21 MRSA isolates had been accrued in order to minimize cost, which equated to a run approximately once per week. Each run contained 21 clinical MRSA isolates and three controls [no template, positive control (MRSA MPROS0386) and negative control (*Escherichia coli* NCTC12241) [10]]. DNA was extracted using the QIAgen DNA mini extraction kit and library preparation was performed using the Illumina Nextera DNA flex kit, as described previously [11]. Sequencing was performed overnight on the Illumina MiniSeq instrument, with a run time of 13 h using the high-output 150 cycle paired-end MiniSeq cartridge and the Generate Fastq workflow. Genomes were required to have the highest match to *S. aureus*; <10 % contamination (<4 % of kmers matching another species in kraken); *mec* gene detected; <30 heterozygous sites >50 bp apart; and a minimum 80 % mapping coverage of the MRSA reference genome (HO 50960412) at a sequence depth of at least 20×.

### Genome analysis

Genome analysis was performed using the automated NGD cloud-based bioinformatics system, as previously described [6,8]. In brief, on completion of the sequencing run this system uploaded raw fastq folders via an encrypted secure upload to the Amazon UK Web Services cloud, where 60 compute nodes were automatically activated. Layers of quality control, read trimming, assembly, mapping and relatedness determination to support transmission detection were automatically performed and genome relatedness was defined between isolates in that run, and with the entire library of previously processed isolates identified during this study. These computations took an average of 35 min for a single run, after which results were downloaded to the NGD database for display and use. Analysis included bacterial species identification, detection of *mec* genes, assignment to STs, identification of pairwise relatedness and use of genetic relatedness to identify clusters. Novel STs were submitted to PubMLST for assignment (https://pubmlst.org). Clonal complexes were determined post-hoc.

### Transmission, acquisitions and outbreaks

We identified genetic clusters [two or more cases (patients and/or healthcare workers) with genetically related MRSA isolates] using a 25 SNP cut-off, determined previously as an inclusive cut-off for recent MRSA transmission [12]. We then sought temporospatial links between patients to identify if and where transmission had occurred within each of the MRSA genetic clusters using a timeline of ward movements and/or clinic visits for hospital in-patients and out-patients, respectively. Epidemiological links were sought between patients from the first day of the study. Ward contact was defined if a case-pair was admitted to the same ward with overlapping dates of admission or within 7 days of each other, as previously described [12]. An out-patient cluster was defined as patients visiting the same clinic on the same day as a known MRSA case. A pseudo-outbreak was defined as patients who had been present on the same ward with overlapping dates of admission but carried MRSA that were not genetically related. Samples from the community were not included in the study, but we sought community transmission for MRSA-positive hospital patients based on a shared GP practice, residential postcode or long-term care facility. No epidemiological or other individual-level information was available to the research team for healthcare workers.

Manual curation of patient timelines and comparison with MRSA relatedness was time consuming and a proof-of-concept NGD user interface was developed during the study and introduced in its last month. This was embedded in the National Health Service (NHS) network through an AIMES environment, a cloud-based safe data environment used by the NHS

(https://www.digitalmarketplace.service.gov.uk/g-cloud/services/598927326333959) and automatically identified and displayed genetically related clusters. Following upload of patient study code and ward movement this interface displayed timelines of ward movements for each patient in a genetic cluster, with the capability to filter information based on ward or patient.

### Impact on infection control decisions

**Fig. 1.**
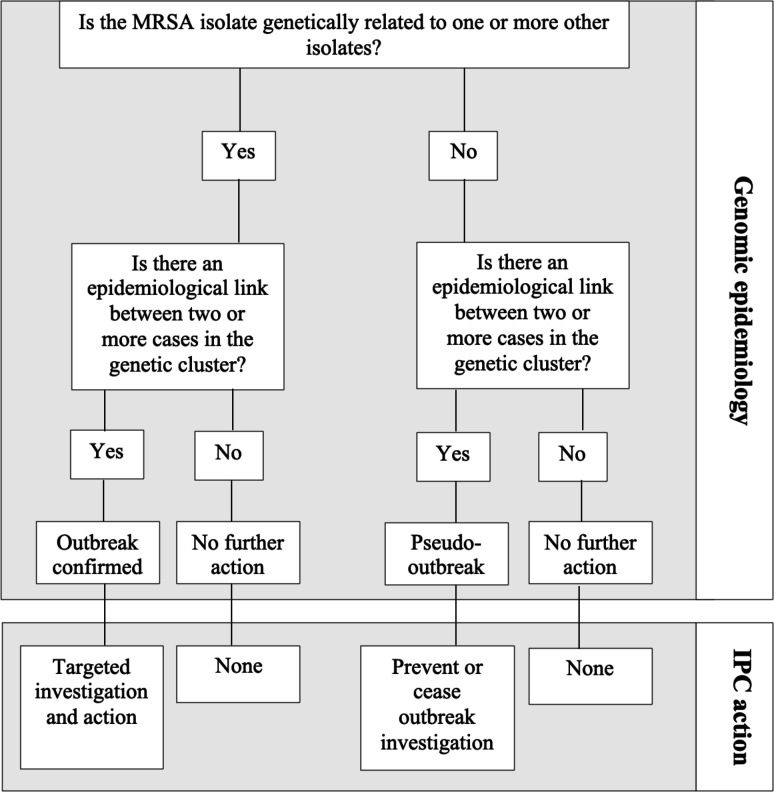
Simplified flow chart summarizing the IPC assessment and classification of genomic epidemiological data. Isolates were defined as related using a 25 SNP cut-off based on pairwise genome comparisons, determined previously as an inclusive cut-off for recent MRSA transmission [12]. Ward contact was defined if a case-pair was admitted to the same ward with overlapping dates of admission or within 7 days of each other, as previously described [12]. An out-patient cluster was defined as patients visiting the same clinic on the same day as a known MRSA case.

Weekly meetings were held between the study team and the hospital IPC team to relay genomic epidemiology findings and record IPC discussions and actions. Fig. 1 shows a simplified flow chart summarizing the IPC assessment and classification of genomic epidemiological data generated by the study. Potential for changes in IPC decision-making were based on outbreak confirmation and targeted investigation and actions (with prevention of onward transmission of MRSA), and identification of pseudo-outbreaks to de-escalate unnecessary IPC investigations and interventions.

## Results

### MRSA patients, and genetic and epidemiological analysis

**Fig. 2.**
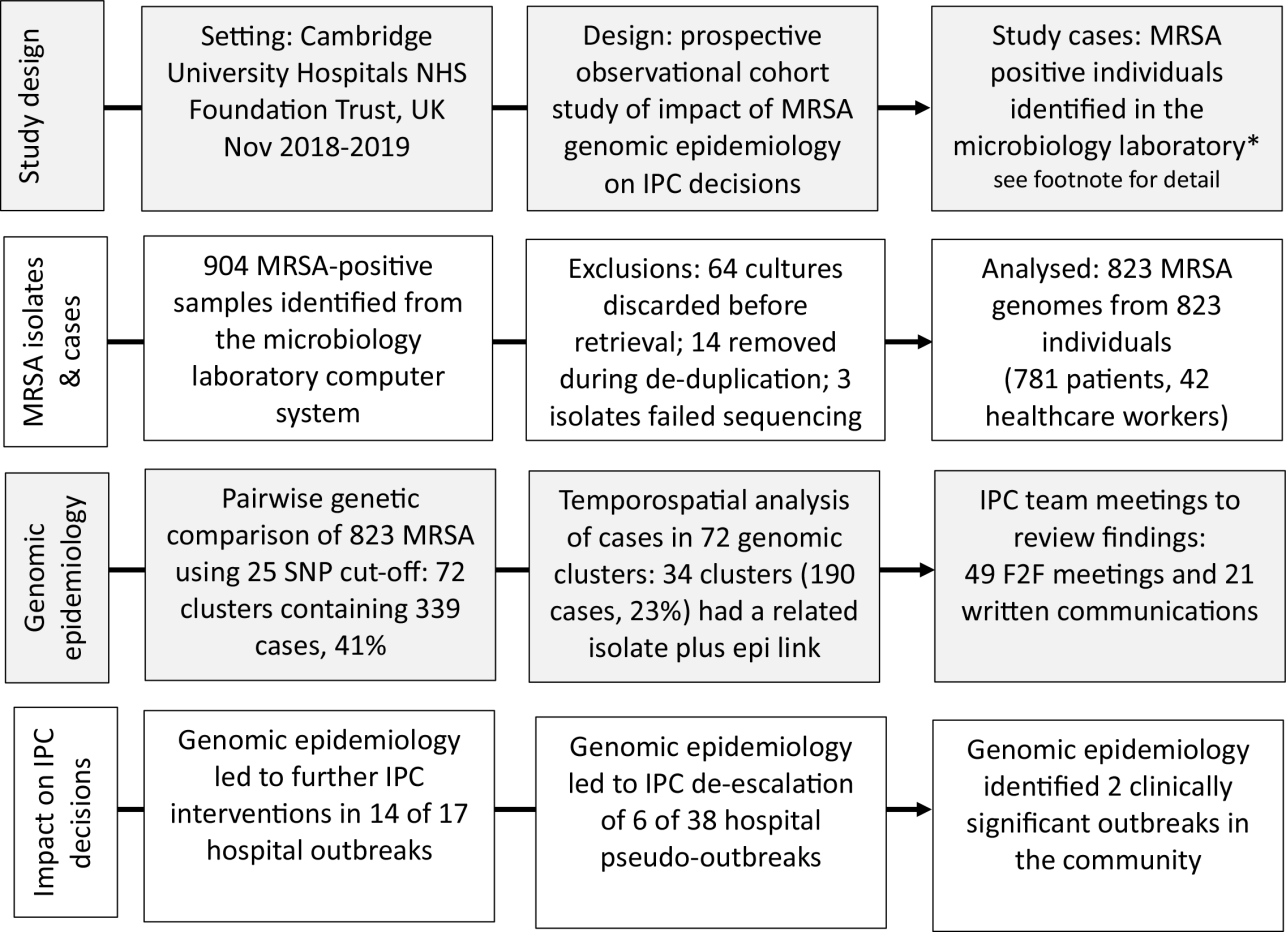
Summary of the study design and main findings. *Included were samples taken from in-patients, out-patients, people reviewed in the Emergency Department and samples taken by the Occupational Health Department from healthcare workers. Excluded were samples submitted by General Practitioners (GPs) and other care providers in the community. F2F, face-to-face.

Fig. 2 summarizes the study design and main findings. We identified 904 MRSA isolates using the hospital IT system over the 12 month study period. Of these, 64 MRSA cultures (7 %) from 64 patients were discarded by the routine microbiology laboratory before they could be retrieved by the study team and were therefore excluded. A further three MRSA isolates from three patients were subsequently excluded for genome data quality reasons post-sequencing. The remaining 837 MRSA isolates from 823 cases were deduplicated and the first MRSA isolate was included in the analysis (i.e. 823 isolates from 823 individuals). Of these, 781 were patients and 42 were healthcare workers at CUH whose samples had been submitted by the Occupational Health department.

Characteristics of the 823 cases and their samples/isolates are shown in Table 1. Just over half were male, and more than a quarter had a past history of MRSA positivity. The majority of positive samples were from MRSA screens, and the most common hospital location where samples were taken was in Emergency Care. The majority of MRSA isolates contained *mecA* (*n*=814, 98.9 %), with a small number (*n*=9, 1.1 %) containing *mecC*. A total of 70 STs were identified, with five STs accounting for 72 % of the total [ST22 (*n*=293, 36 %); ST59 (*n*=91, 11 %); ST45 (*n*=85, 10 %); ST5 (*n*=63, 8 %); ST1 (*n*=57, 7 %)]. Further isolate details are provided in Table S1.

**Table 1.** Characteristics and demographics of 823 MRSA-positive study participants

Characteristics	No. (%)
Age, years (median, IQR)	61, 30–80
Gender, male	422 (51.3)
Known previously to be MRSA positive	229 (27.8)
Place of residence on admission	
Own home	704 (85.5)
Long-term care facility	113 (13.7)
Homeless	6 (0.7)
Location where sample taken	
Emergency care	323 (39.3)
Clinic	204 (24.8)
Hospital ward	196 (23.8)
Day ward	58 (7.1)
Occupational health	42 (5.1)
Type of sample	
MRSA screen	688 (83.6)
Clinical – non-sterile site	93 (11.3)
Clinical – sterile site	42 (5.1)

IQR, interquartile range.

We conducted a genomic analysis to identify genetically related MRSA clusters using a pairwise genome comparison and applying a 25 SNP cut-off after each sequencing run. We then used temporospatial clustering to identify where transmission had occurred within the MRSA clusters for discussion each week with the IPC team. This analysis was performed for every sequencing run (approximately weekly) with rolling results over time provided during face-to-face meetings. For simplicity, the genomic epidemiology and IPC information gathered from weekly meetings between the research and IPC teams are summarized here for the entire study period.

Genomic analysis identified 72 clusters of MRSA containing two or more isolates, which incorporated 339 (41 %) cases (Table S2). Temporospatial analysis of these clusters resulted in detection of a link between two or more people in 34 of these MRSA clusters [190 (23 %) cases], of which 13 clusters contained cases with hospital links alone [eight clusters, 47 (6 %) cases] or hospital plus community links [five clusters, 39 (5 %) cases]. The remainder had putative temporospatial links in the community based on GP and/or postcode, suggesting transmission prior to admission.

Thirteen of the 42 isolates from healthcare workers resided in clusters. This identified three pairs of healthcare workers with MRSA isolates that were related to each other but not to patient isolates, while the remaining seven isolates were assigned to six different clusters that also contained patients. MRSA-positive healthcare workers were managed by the Occupational Health Department.

We observed a single transmission event in 11/13 hospital clusters, but two MRSA clusters were more complex (Table 2). MRSA cluster CUH0095 (seven isolates) contained two discrete transmission events in time, on different bays of the same ward. MRSA cluster CUH0006 (66 isolates) contained four discrete transmission events containing two, three, two and five patients, respectively (the remaining isolates in the cluster not having a detectable epidemiological link). The first three transmission events happened on three different wards. The fourth transmission event containing five patients occurred in an out-patient clinic. We also identified an additional out-patient cluster (CUH0003) linked to the diabetic foot clinic. We defined these as 17 unique hospital transmission events.

**Table 2.** Action review for 17 MRSA transmission events where at least two patients in a genetic cluster had hospital links

Cluster ID	Patients with ward epidemiological link	Patients with clinic epidemiological link	Investigated by IPC team	Patient screening for MRSA	Staff screening for MRSA	Ward clean/PPE and/or hand hygiene audit	Outbreak meeting
CUH0136	3	0	Yes				
CUH0049	2	0	Yes				
CUH0094	3	0	Yes			Yes	
CUH0125	2	0	*				
CUH0118	2	0	Yes				
CUH0120	3	0	Yes	Yes			
CUH0102	2	0	Yes			Yes	
CUH0092	2	0	*				
CUH0056	3	0	Yes	Yes			
CUH0012	2	0	Yes	Yes			Yes
CUH0095_A	2	0	Yes	Yes			
CUH0095_B	2	0	Yes	Yes	Yes		Yes
CUH0006_A	2	0	Yes				
CUH0006_B	3	0	Yes	Yes			
CUH0006_C	2	0	Yes				
CUH0006_D	0	5	†				
CUH0003	0	9	Yes			Yes	Yes

*Action was already underway by the time of the IPC meeting for clusters 125 and 092.

#†Action was already underway due to a separate outbreak in the same clinic

Letters of the alphabet are used to designate where more than one outbreak occurred in association within the same genetic cluster. PPE, personal protective equipment.

We sought evidence for pseudo-outbreaks (where MRSA-positive patients had temporospatial clustering but isolates were not related). We identified 38 instances where two or more in-patients carried unrelated isolates but shared a ward location during overlapping dates. Around half of instances (*n*=20) involved just two MRSA patients, but the remaining 18 events involved between three and eight patients. Specifically, pseudo-outbreaks involving three patients occurred eight times; involving four patients occurred four times; involving five patients occurred five times; and involving eight patients occurred once.

Six pseudo-outbreaks contained ST22 isolates only and multi-locus sequence typing (MLST) alone mis-classified these as outbreaks. For 20 pseudo-outbreaks, every isolate in the cluster was a different ST and MLST would have correctly classified these. Twelve pseudo-outbreaks were more complex – more than one ST was involved, but each cluster also contained two or more isolates of the same ST. For example, one pseudo-outbreak of five cases involved four ST22 isolates and one ST59 isolate. Another pseudo-outbreak of five cases involved four ST22 isolates and one ST1 isolate. In these circumstances, MLST would not have been able to exclude an outbreak. Based on these data, we suggest that 18/38 pseudo-outbreaks would not have been confirmed based on MLST data.

### Interactions with the IPC team

Research results were available to the IPC team on the day that genomic epidemiological data for a given sequencing run were generated. The study and IPC teams interacted through 49 face-to-face weekly meetings and 21 written communications made outside of the weekly meeting cycle. The IPC team are hospital-based and discussions focused largely on the 17 transmission events with one or more epidemiological links to the hospital. A summary of actions that arose from these communications are shown in Table 2. Genomic epidemiological information led directly to further investigation by the infection control team in 14 of these clusters. Actions included targeted MRSA screening of direct patient contacts if only single transmission events were identified, or screening of all patients on a ward and healthcare workers if further transmission was identified. Beds within affected bays were usually closed to new admissions while this was undertaken unless the need for urgent clinical care prevented this. The IPC team performed audits of IPC practice on the wards and provided additional education where necessary. Outbreak investigations were coordinated by formal incident management team meetings where on-going MRSA transmission was identified. All of these interventions are resource intensive.

Sequence data made available to the IPC were also evaluated in the context of the 38 instances where patients carried unrelated isolates but were temporospatially clustered. The genomic data enabled de-escalation of IPC interventions in six of these pseudo-outbreaks. Formal incident management team meetings were unnecessary and additional patient and staff MRSA screening was not required. Bed closure was avoided. The six instances that were de-escalated involved five patients (on three separate occasions), and four, three or two patients (one occasion each).

The study focused on in-hospital MRSA transmission and did not include MRSA-positive samples taken in the community, but we identified two significant community outbreaks involving patients who subsequently presented to hospital. Cluster CUH0111 (seven MRSA isolates/patients) involved people residing in the same nursing home. The hospital-based IPC team informed the community nurse each time there was a new MRSA-positive sample from a resident of this facility, who in turn fed this information back to the facility. Cluster CUH0012 (12 MRSA isolates/patients) included two isolates from bloodstream infections that were identified early in the study. Further investigation found that several patients in the cluster were intravenous drug users or from the homeless community, and were registered at the same GP practice. The community health protection team and GP practice were informed, and several meetings were held to identify relevant action. This included enhanced surveillance for MRSA in lesions or wounds on individuals from the homeless/intravenous drug use community who re-presented to the Emergency Department.

## Discussion

There is growing evidence for the superiority of genomic epidemiology compared with standard IPC investigations in confirming and refuting transmission and outbreaks caused by numerous pathogens including those where detection of transmission is otherwise highly challenging [13,14]. This is most powerful when undertaken routinely for specified pathogens such as MRSA and multidrug-resistant (MDR) Gram-negative species, rather than retrospectively to inform a standard IPC investigation. This capability is further enhanced when combined with machine learning using the electronic health record to identify transmission routes [15,16]. Together, this has been shown to detect multiple outbreaks not identified using traditional IPC methods, and to correctly identify the transmission routes for most outbreaks [15]. It also corrects misidentified outbreaks where transmission did not occur.

These innovations would be predicted to lead to substantial cost savings and enhance patient safety, and evidence for this is growing. A mathematical modelling study of cost effectiveness of pro-active genomic surveillance of MRSA reported a cost benefit to the healthcare system [9]. Methods have also been reported for the economic evaluation of bacterial whole genome sequencing surveillance compared to standard of care in detecting hospital outbreaks [9,17]. A budget impact analysis of routinely using whole-genomic sequencing of six pathogenic MDR bacterial species was performed in Queensland, Australia [18]. This concluded that compared with standard of care, genomic surveillance at a state-wide level could stop a substantial number of hospital patients from being infected with MDR organisms, prevent deaths and save healthcare costs.

Routine genomic epidemiology for MDR pathogens is increasingly implemented in healthcare settings, evaluation of which has shown that this is feasible and can enable targeted IPC interventions [19]. The study described here adds further evidence for the value of its clinical implementation. Predictably, we showed that the combination of sequence data and temporospatial analysis led to the detection of otherwise undetected outbreaks and identified pseudo-outbreaks. Both of these categories led to specific actions by the hospital IPC team, with a more targeted use of IPC time and resources focused on true outbreaks and de-escalation of MRSA clusters that had occurred by chance. This demonstrates that the approach makes a tangible difference to prospective decisions involving patient care in a study that reflected a practical and real-world study design.

We identified 38 instances where two or more in-patients carried unrelated isolates but shared a ward location during overlapping dates. While it was positive to report that genomic data enabled de-escalation of IPC interventions in six of these pseudo-outbreaks, IPC management was not altered for the majority. There were several contributing factors to this relatively low rate of conversion to IPC action. More than half of pseudo-outbreaks (*n*=20) involved just two patients and were generally self-limiting. In some cases, ward screening had already been triggered and no further cases had been found so the genomics data did not have a tangible effect on IPC actions. The turnaround time for sequencing was also a contributory factor to the likelihood of IPC response to outbreaks and pseudo-outbreaks.

The detection of outbreaks beyond the hospital setting is an additional advantage for genomic epidemiology, both in care homes and other residential facilities to optimize their own IPC, and more broadly for public health. Detecting two significant community MRSA outbreaks went beyond the original focus of the study but generated information that led to actions aiming to curtail further extension. The responsibility for IPC is often place-based and focused on either hospitals or the community, even though this represents a continuum for patient care. A previous 12 month prospective genomic epidemiology study at our hospital of 1465 individuals with at least one MRSA-positive sample processed by our laboratory, including community samples, revealed 173 transmission clusters [4]. Of these, 27 clusters (72 people) involved community contacts alone and 28 clusters (157 people) had both hospital and community contacts. This suggests that genomic epidemiology could generate information that would support a more holistic IPC framework.

Our study has several limitations. Our genomic epidemiological analysis began on the study start date, and the inclusion of data in the weeks and months preceeding this may have identified further links between patients. By excluding samples taken in the community, the number of patients presenting to hospital who were found to be MRSA positive as a result of community transmission will be under-represented. Our study only benefited from automated analysis of epidemiological links towards the end of the study, which is a vital component for efficient and real-time analysis. We performed a sequencing run each time that 21 MRSA isolates had been identified. The generation of daily outputs would be predicted to have a greater impact on IPC decisions, and could be achieved using alternative sequencing technologies that support cost-effective single isolate sequencing. We did not quantify cost savings for the NHS, or model the number of putative instances of onward MRSA transmission that were averted.

In conclusion, this study has reinforced the benefit of adding genome data to epidemiological data collected by IPC teams in detecting and refuting MRSA outbreaks, and goes further in providing a quantification of the impact of genomic epidemiology in routine IPC practice.

## supplementary material

10.1099/mgen.0.001235 Uncited Table S1.

## Abbreviations

CUH: Cambridge University Hospitals NHS FoundationTrust
F2F: Face-to-face
GP: general practitioner
IPC: infection prevention and control
MDR: multidrug-resistant
MLST: multilocus sequence typing
MRSA: methicillin-resistant *Staphylococcus aureus*
NGD: Next Gen Diagnostics
NHS: National Health Service
PHE: Public Health England
SNP: Single nucleotide polymorphism
ST: sequence type
